# L-Lactate Treatment at 24 h and 48 h after Acute Experimental Stroke Is Neuroprotective via Activation of the L-Lactate Receptor HCA_1_

**DOI:** 10.3390/ijms25021232

**Published:** 2024-01-19

**Authors:** Samuel J. Geiseler, Alena Hadzic, Marvin Lambertus, Karl Martin Forbord, Ghazal Sajedi, Arthur Liesz, Cecilie Morland

**Affiliations:** 1 Section for Pharmacology and Pharmaceutical Biosciences, Department of Pharmacy, University of Oslo, 0316 Oslo, Norway; alena.hadzic@farmasi.uio.no (A.H.); marvin.lambertus@liu.se (M.L.); k.m.f.forbord@farmasi.uio.no (K.M.F.); s.g.sajedi@studmed.uio.no (G.S.); 2 Institute for Stroke and Dementia Research, Klinikum der Universität München, Ludwig-Maximilians University Munich, 81377 Munich, Germany; arthur.liesz@med.uni-muenchen.de; 3 Graduate School of Systemic Neurosciences Munich, 82152 Munich, Germany; 4 Munich Cluster for Systems Neurology (SyNergy), 81377 Munich, Germany

**Keywords:** stroke, dMCAO, vascularization, angiogenesis, GPR81, HCAR1, HCA_1_, exercise, L-lactate

## Abstract

Stroke is the main cause for acquired disabilities. Pharmaceutical or mechanical removal of the thrombus is the cornerstone of stroke treatment but can only be administered to a subset of patients and within a narrow time window. Novel treatment options are therefore required. Here we induced stroke by permanent occlusion of the distal medial cerebral artery of wild-type mice and knockout mice for the lactate receptor hydroxycarboxylic acid receptor 1 (HCA_1_). At 24 h and 48 h after stroke induction, we injected L-lactate intraperitoneal. The resulting atrophy was measured in Nissl-stained brain sections, and capillary density and neurogenesis were measured after immunolabeling and confocal imaging. In wild-type mice, L-lactate treatment resulted in an HCA_1_-dependent reduction in the lesion volume accompanied by enhanced angiogenesis. In HCA_1_ knockout mice, on the other hand, there was no increase in angiogenesis and no reduction in lesion volume in response to L-lactate treatment. Nevertheless, the lesion volumes in HCA_1_ knockout mice—regardless of L-lactate treatment—were smaller than in control mice, indicating a multifactorial role of HCA_1_ in stroke. Our findings suggest that L-lactate administered 24 h and 48 h after stroke is protective in stroke. This represents a time window where no effective treatment options are currently available.

## 1. Introduction

Cerebral stroke is the second leading cause of death worldwide [1] and one of the most prevalent causes of long-term disabilities [2]. Acute ischemic stroke is caused by an occlusion of a cerebral blood vessel, normally by a thrombus, causing an interruption of blood supply to the downstream brain tissue [3,4,5]. This form of stroke accounts for about 87% of all stroke cases [6]. Time is of the essence when it comes to stroke treatment: within minutes after a halt in the blood supply, neural cells start to die. Hence, a rapid re-establishment of blood flow is highly important to prevent brain damage (for review, see [7]). Several mechanisms are at play in the early phases of stroke, including hypoxia, ischemia, excitotoxicity, oxidative stress, and neuroinflammation, all of which contribute to cell death.

Current treatments, such as thrombolysis and thrombectomy, are highly effective but can only be used in a subset of patients and are restricted to a short period after stroke onset [8]. Only about 5% of all patients with ischemic stroke meet the criteria for thrombolysis [9], and hence, most strokes go largely untreated. To reduce the number of patients who experience disabilities after stroke, novel treatment options—with more extended time windows—would be highly appreciated.

Intrinsic repair mechanisms are induced in the brain in response to stroke [10], and therapies aiming at enhancing these mechanisms may prove to be efficient in the treatment of stroke. For example, increased angiogenesis has been shown after stroke in humans [11] as well as after prolonged hypoxia in rodents [12,13]. The increased density of capillaries resulting from angiogenesis may protect the penumbra and reduce the lesion volume. Enhanced neurogenesis in the subventricular zone and the subgranular zone has also been reported in response to stroke in animal models [9]. Interestingly, several of the pro-inflammatory cytokines and growth factors released in response to stroke have detrimental effects in the early phase after stroke but may enhance repair at later stages. An example of this is vascular endothelial growth factor A (VEGFA) which causes permeability of the blood–brain barrier, thereby worsening brain edema in the early phase, yet is a potent inducer of angiogenesis at later phases (reviewed in [14]). Similarly, the pro-inflammatory cytokine interleukin (IL)-6 propels the immune response at early stages of stroke but contributes to neuroprotection and neurogenesis during stroke recovery [15,16,17]. Different therapeutic approaches to regulate the inflammatory response, the levels of growth factors, or other signaling molecules released in response to stroke, have been tested in animal models. Translation into human therapy has, however, been challenged by the intricacy and temporal changes in the signaling cascades these therapies aim to affect. So far, no neuroprotective therapy has been approved for stroke treatment.

L-lactate increases in the brain in response to stroke, and this increase is presumably proportional to the size and severity of the infarction. Somewhat contra-intuitively, exogenously L-lactate delivered either intracerebroventricularly or systemically at the time of reperfusion has previously been shown to reduce the lesion size in mice [18,19]. The exact mechanism(s) underlying the beneficial effects of L-lactate injections in stroke remains to be elucidated. Emerging evidence demonstrates that L-lactate affects brain cells as a metabolite and by activating the G protein-coupled receptor hydroxycarboxylic acid receptor 1 (HCA_1_; previously known as GPR81 or HCAR1) [20]. For instance, we have previously reported that activation of HCA_1_ induced angiogenesis in the hippocampus and the neocortex of mice [21]. Furthermore, we have demonstrated that HCA_1_ activation also regulates neurogenesis in the ventricular–subventricular zone [22]. Both angiogenesis and neurogenesis are potentially important contributors to neuroprotection and/or tissue regeneration after stroke, but whether HCA_1_-mediated mechanisms are involved in neuroprotection after stroke has not been investigated.

## 2. Results

### 2.1. Lactate Treatment Reduced the Lesion Volume Three Weeks after Stroke Induction in Wild-Type Mice but Not in HCA_1_ Knockout Mice

**Figure 1:**
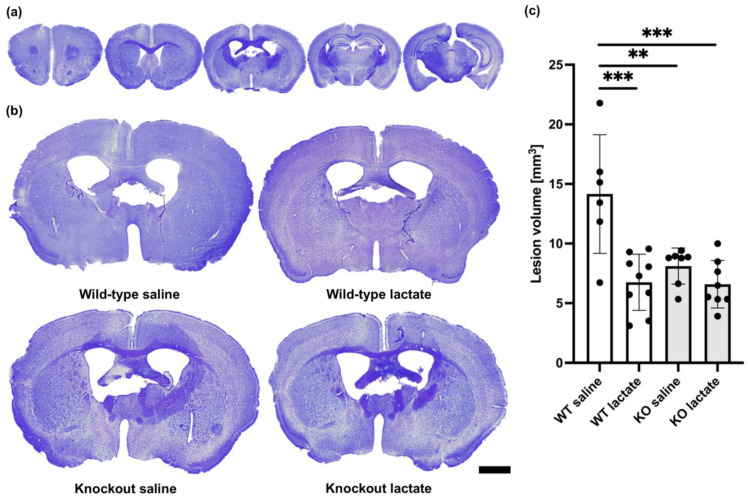
Lesion volumes at three weeks after stroke were reduced in a hydroxycarboxylic acid receptor 1 (HCA_1_)-dependent manner by lactate treatment. (**a**) Representative cresyl violet (CV)-stained coronal brain sections ranging from 1.5 mm rostral to 2.5 mm dorsal of bregma. Only 5 out of 25 analyzed sections per animal are shown here. (**b**) Larger images of representative CV-stained sections taken 0.5 mm dorsal to bregma from wild-type mice treated with either saline (control group; top left) or lactate (top right), or HCA_1_ knockout mice treated with saline (down left) or lactate (down right) at three weeks after induction of distal medial cerebral artery occlusion (dMCAO). (**c**) Quantitative assessment of the lesion volumes in WT mice (white bars) and HCA_1_ KO mice (grey bars) three weeks after treatment with saline or lactate. The black dots represent individual mice. ** *p* < 0.01; *** *p* < 0.001; scale bar = 1 mm.

At three weeks after a permanent occlusion of the left distal medial cerebral artery (dMCA), a cortical atrophy of the left hemisphere was observed (Figure 1). This cortical atrophy was evident as a shorter distance between the corpus callosum and the brain surface. In some sections, we observed areas with smaller and more densely packed nuclei. These areas generally appeared more lightly stained and probably represented areas where neuronal death and gliosis had occurred. We also observed darker-appearing areas, presumably representing scarred tissue. When calculating the total lesion volume, these areas were added to the area of the atrophic tissue. At three weeks after dMCA occlusion (dMCAO), there was an HCA_1_-dependent reduction in the lesion volume in response to L-lactate treatment (Figure 1): the average lesion volume in the wild-type (WT) mice treated with L-lactate (6.75 ± 2.36 mm^3^, n = 9) was smaller than the lesion volume in WT mice treated with saline (14.15 ± 4.98 mm^3^, n = 6) (*p* < 0.001; one-way ANOVA, Tukey’s post hoc test). This effect of L-lactate was not found in the knockout (KO) mice: the lesion volume measured in KO mice treated with L-lactate (6.59 ± 1.99 mm^3^, n = 8) was not different from the lesion volume in KO mice treated with saline (8.11 ± 1.52 mm^3^, n = 7) (*p* = 0.598; one-way ANOVA, Tukey’s post hoc test). Interestingly, both groups of KO mice had smaller lesion volumes than the WT control group (*p* < 0.01 for both comparisons; one-way ANOVA, Tukey’s post hoc test, SPSS).

### 2.2. At One Week after dMCAO, the Lesion Volumes Were Unaffected by the Treatments or Genotypes

**Figure 2:**
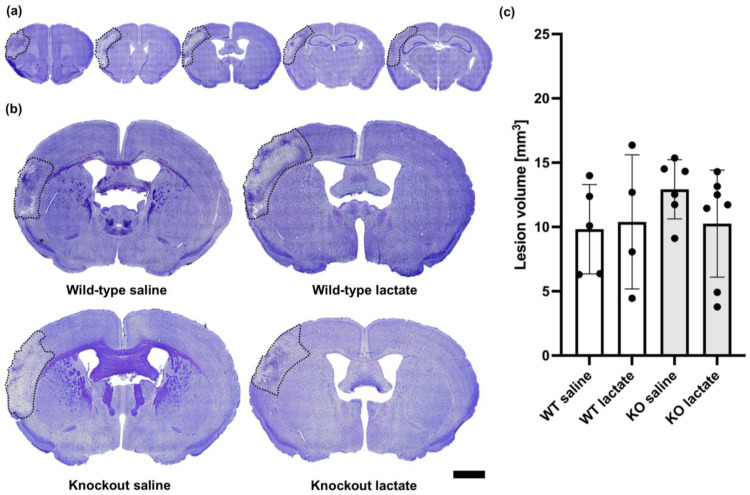
Lesion volumes at one week after stroke were unaffected by lactate treatment and the presence of hydroxycarboxylic acid receptor 1 (HCA_1_). (**a**) Representative CV-stained coronal brain sections ranging from 1.5 mm rostral to 2.5 mm dorsal of bregma. Only 5 out of 25 analyzed sections per animal are shown here. (**b**) Larger images show representative CV-stained sections taken 0.5 mm dorsal to bregma from wild-type mice treated with either saline (control group; top left) or lactate (top right), or HCA_1_ knockout mice treated with saline (down left) or lactate (down right) at one week after induction of distal medial cerebral artery occlusion (dMCAO). The lesion area is outlined (black dotted line) for visualization. (**c**) Quantitative assessment of the lesion volumes in WT mice (white bars) and HCA_1_ KO mice (grey bars) one week after treatment with saline or lactate. The black dots represent individual mice. Scale bar = 1 mm.

At one week after dMCAO surgery, the lesions were visible and infarcted and healthy tissue was easy to distinguish (Figure 2). The infarcted tissue appeared lighter and contained fewer nuclei than the healthy tissue. In the core of the infarction, a darker area was sometimes present, probably representing glial scarring or fibrosis. As opposed to what was seen after three weeks, at one week after dMCAO, there were no significant differences in lesion volumes between the groups (*p* = 0.514; one-way ANOVA). The lesion volumes measured one week after stroke induction were as follows: WT saline: 9.83 ± 3.48 mm^3^ (n = 5); WT lac: 10.39 ± 5.22 mm^3^ (n = 4); KO saline: 12.93 ± 2.30 mm^3^ (n = 6); and KO lactate 10.26 ± 4.16 mm^3^ (n = 7). All stroke-operated groups had lesion volumes that were much larger than in sham animals (1.95 ± 0.97 mm^3^, n = 3).

### 2.3. L-Lactate Treatment Increased Capillary Density Three Weeks after dMCAO in WT Mice, but Not in HCA_1_ KO Mice

**Figure 3:**
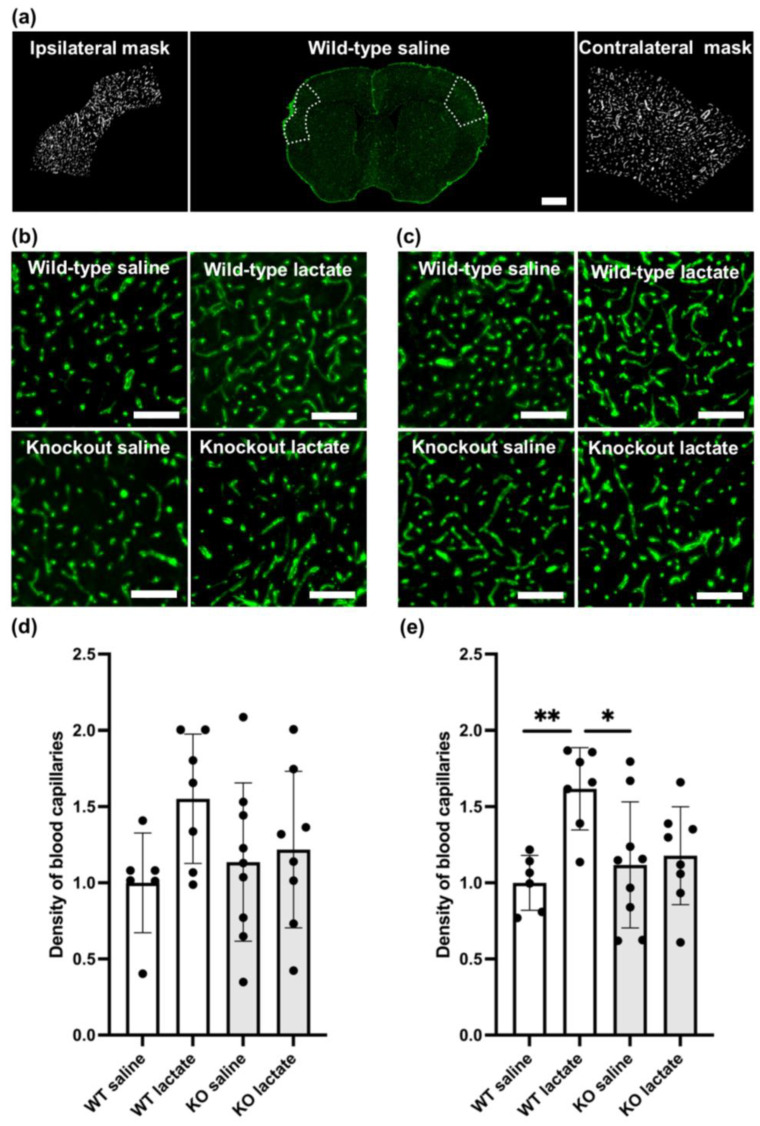
Angiogenesis three weeks after stroke was increased in a hydroxycarboxylic acid receptor 1 (HCA_1_)-dependent manner by lactate treatment. (**a**) Coronal section immunolabeled for the basal lamina marker collagen IV (green) in the middle; the selections used to quantify capillary densities are shown by the white dotted lines, and the resulting masks are shown on each side. (**b**) Representative confocal images of capillaries in the ipsilateral cortex of all treatment groups. (**c**) Representative confocal images of capillaries in the contralateral cortex of all treatment groups. (**d**) Quantitative assessment of the capillary densities of the ipsilateral cortex of WT mice (white bars) and HCA_1_ KO mice (grey bars) after treatment with saline or lactate. Numbers represent the area covered by capillaries divided by the area of the region of interest (ROI; area covered by larger vessel subtracted) and are normalized to the average capillary density of the WT control (mean ± SD). (**e**) Quantitative assessment of the capillary densities of the contralesional cortex of WT mice (white bars) and HCA_1_ KO mice (grey bars) after treatment with saline or lactate. Numbers represent the area covered by capillaries divided by the area of the region of interest (area covered by larger vessel subtracted) and are normalized to the average capillary density of the WT control (mean ± SD). The black dots represent individual mice. * *p* < 0.05; ** *p* < 0.01; scale bars = 1 mm (**a**) and 125 µm (**b**,**c**).

On the contralesional side, WT mice treated with L-lactate showed increased capillary density compared to saline-treated WT (*p* = 0.01; one-way ANOVA, Tukey’s post hoc) or KO mice (*p* = 0.023; one-way ANOVA, Tukey’s post hoc). Compared to L-lactate-treated KO mice, L-lactate-treated WT mice had only marginally higher densities of capillaries (*p* = 0.062; one-way ANOVA, Tukey’s post hoc; Figure 3). This difference was not evident in the ipsilateral tissue surrounding the stroke area (*p* = 0.186; one-way ANOVA; Figure 3). The exact values (contralesional side, mean ± SD capillary coverage in percent, normalized to WT saline) were as follows: WT saline: 1.00 ± 0.18 (n = 6); WT lac: 1.6 ± 0.22 (n = 7); KO saline: 1.18 ± 0.41 (n = 9); KO lactate 1.23 ± 0.32 (n = 8).

### 2.4. At One Week after dMCAO, the Capillary Density Was Unaffected by the Treatments or Genotypes

Similar to lesion size, neither L-lactate injections nor genotype seemed to affect capillary density one week after induction of stroke (Figure S1). There was no difference in capillary density between the different treatments or genotypes, neither on the contralesional side (*p* = 0.786, one-way ANOVA) nor on the ipsilateral side surrounding the stroke (*p* = 0.900, one-way ANOVA). The contralesional capillary coverage in percent of the ROI (mean ± SD), normalized to WT saline, were as follows: WT saline: 1.00 ± 0.15 (n = 8); WT lac: 1.07 ± 0.39 (n = 4); KO saline: 1.06 ± 0.28 (n = 6); KO lactate 0.93 ± 0.28 (n = 6).

### 2.5. Neurogenesis Was Unaffected by the Treatments or Genotypes Both at Three Weeks and One Week after dMCAO

**Figure 4:**
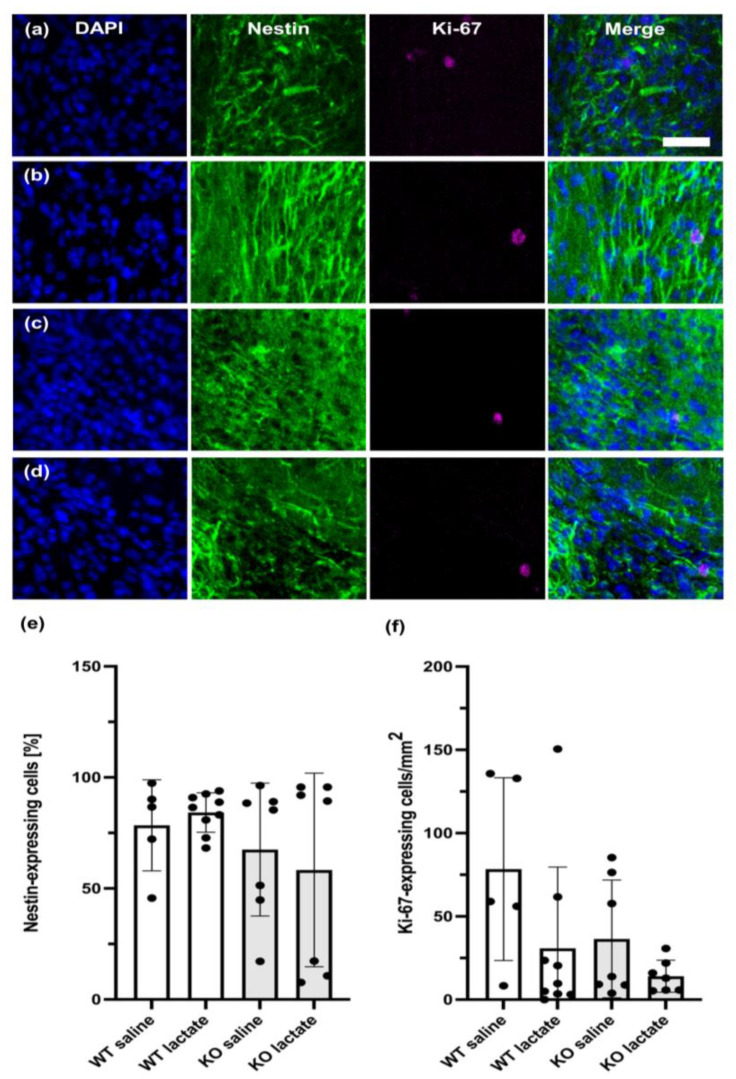
Neurogenesis at three weeks after stroke was unaffected by lactate treatment and the presence of hydroxycarboxylic acid receptor 1 (HCA_1_). Neurogenesis or neural migration observed at three weeks after stroke was not affected by lactate treatment or the presence of HCA_1_. Coronal section immunolabeled with markers for neuroprogenitor cells (nestin; green), proliferating neuroblasts (Ki-67; magenta), and nuclei (DAPI; blue), of WT mice treated with (**a**) or saline or (**b**) L-lactate, and HCA_1_ KO mice treated with (**c**) saline or (**d**) L-lactate. (**e**,**f**) Quantitative assessment of the neuroprogenitor cells (**e**) and proliferating neuroblasts (**f**) of WT mice (white bars) and HCA_1_ KO mice (grey bars). The black dots represent individual mice. Scale bar = 33 µm.

No significant difference in the perilesional neurogenesis markers nestin and Ki-67 were found between any of the groups, neither three week (Figure 4) nor one week (Figure S2) after stroke induction.

## 3. Discussion

### 3.1. HCA_1_-Dependent Neuroprotective Effect of L-Lactate

The halving of the lesion volume observed in response to L-lactate treatment in WT mice was not reproduced in the HCA_1_ KO mice. This strongly suggests that the neuroprotective effect was mediated through HCA_1_ activation. The lesion volumes, measured as lost, dead/dying and unhealthy tissue combined, were smaller three weeks after stroke than one week after stroke, except for in the WT control. This suggests that the effect of L-lactate via HCA_1_ involves repair and/or protection of the penumbra and not the acute neurotoxic effects of the stroke. Importantly, the lactate treatment was administered at 24 h and 48 h after stroke induction, which represents a time window where conventional treatment options are lacking.

Accompanying the reduced atrophy in response to stroke in WT mice treated with L-lactate was an HCA_1_-dependent increase in capillary density. In the perilesional cortex, this did not reach statistical significance, presumably because the small size of this area introduces more variability. In the intact contralateral cortex, however, the capillary density was increased in WT mice after L-lactate treatment compared to WT controls. In HCA_1_ KO mice, L-lactate did not increase capillary density. This indicates a causal link between HCA_1_ activation and cortical angiogenesis, which likely contributes to reduced lesion volumes. The mechanisms linking HCA_1_ activation to angiogenesis likely involve an increase in VEGFA levels [14], but whether HCA_1_ activation also affects the inflammatory response has not been investigated. 

Mice from the two genotypes did not differ in body weight (WT: 25.7 ± 5.0 g, KO: 26.5 ± 4.6 g), and hence, the difference in the lesion volumes between WT and KO mice did not represent a noticeable difference in risk factors like obesity, insulin resistance, etc. For the same reason, the differential effect of L-lactate between the genotypes could not be ascribed to differences in dosage volume, distribution volumes or other pharmacokinetic aspects related to a difference in body composition. The lactate injections used in the present study have been reported to cause plasma lactate to increase to about 10 mmol/L [21], which is within the physiological range. We have previously verified that the dose of L-lactate used in the present study did not cause anxiety-related behavior in the “elevated zero-maze” [21], which could otherwise be a confounding factor. We therefore conclude that L-lactate leads to reduced atrophy after stroke by activation of HCA_1_-dependent mechanisms.

### 3.2. A Two-Sided Effect of HCA_1_ in Stroke Protection

An intriguing finding in this study is that the genetic absence of HCA_1_, independently of L-lactate treatment, resulted in reduced lesion volumes compared to control mice: both groups of KO mice developed smaller lesion volumes than the WT control group. Similar observations suggesting genetic compensation in response to gene knockout have been made before (for review, see [23]), suggesting that the lack of HCA_1_ may induce compensatory changes. As an alternative mechanism, Shen et al. have demonstrated that inhibition of HCA_1_ with the antagonist 3-hydroxy-butyrate (3-OBA) was protective in transient MCAO [24]. However, these results are not straightforward to interpret since 3-OBA not only acts on HCA_1_ but is also the main endogenous agonist at HCA_2_ [25], which in itself has been shown to have a neuroprotective effect in the ischemic brain [26]. 

### 3.3. The Stroke Model

The dMCAO model used in the present study results in a lesion within the cortex and covers about 10–15% of the hemisphere, thereby mimicking most human stroke lesions located in the cortical MCA territory [27,28,29]. As a permanent occlusion model, the dMCAO mimics a stroke without spontaneous or treatment-induced recanalization. For the current study, it was essential to model untreated strokes, as we aimed to investigate whether L-lactate, through HCA_1_-activation, could represent a treatment for patients who were not eligible for thrombolysis or thrombectomy. Furthermore, only about 3.7–9% of all large-vessel strokes in the US obtain recanalization. Therefore, the permanent dMCAO is now the model recommended in the “standards regarding preclinical neuroprotective and restorative drug development, including Stroke” (STAIR) [30], and is becoming increasingly acknowledged by the research community [31,32].

### 3.4. L-Lactate

The L-lactate solution in the present study is hypertonic, while the saline is isotonic. Treatment with hypertonic solutions, normally mannitol or hyperosmolar saline, are beneficial in stroke, as they reduce the development of edema by retracting water in the circulation instead of in the brain (for review, see [33]). In the current study, however, L-lactate or saline were injected intraperitoneally (i.p.). The injected substance would, therefore, require uptake from the ascites fluid before entering the systemic circulation. Any osmotic effects of the injected L-lactate would likely affect the abdominal organs prior to uptake and not the brain. The finding that L-lactate reduced the stroke volume in WT animals but not in HCA_1_ KO animals supports this line of thought. An osmotic effect of L-lactate would presumably have prevented brain edema in both genotypes equally. 

### 3.5. Potential for Translation into the Treatment of Stroke Patients

Our results confirm the effects reported by Berthet et al. [18], who demonstrated that L-lactate injection was neuroprotective when given at the time of reperfusion after a transient MCAO. Although using a different stroke model (transient MCAO in the study by Berthet et al. vs. permanent dMCAO in the present study), different routes of administration (intracranial and intravenous in the study by Berthet et al. vs. intraperitoneal in the present study), different doses of L-lactate (0.2 µmol injected intracerebroventricularly; 1 mmol/kg injected intravenously vs. 18 mmol/kg injected intraperitoneally), and a different time window (one injection at the time of reperfusion in the study by Berthet et al. vs. two injections, at 24 h and 48 h, in the present study), the two studies still demonstrate a similar protective effect of L-lactate in stroke. This suggests that the effect of lactate in stroke treatment is robust, which is essential for translating these findings into humans. Our data, however, introduce an extended timeframe where L-lactate treatment can benefit the treatment after a stroke. Furthermore, identifying HCA_1_-activation as the mechanism through which L-lactate increases angiogenesis and reduces the lesion after stroke opens the development and testing of more specific and potent HCA_1_ agonists that may be used as stroke therapeutics in the future. Some agonists are already in use in laboratory research [34,35], but data on their safety and bioavailability in vivo, including the ability to cross the blood–brain barrier, are lacking. In the present study, L-lactate treatment was tested as monotherapy. Further investigations may reveal if HCA_1_ agonists are beneficial as monotherapies in human ischemic stroke. Given the multifactorial pathophysiology of stroke, one possibility would also be to test the efficacy of HCA_1_ agonist in combination with drugs targeting other mechanisms. The finding that L-lactate induced angiogenesis [21] opens the possibility to test the therapeutic potential HCA_1_ activation in other neurological diseases where hypoperfusion is part of the pathophysiology; one possibility in this respect is dementia, including Alzheimer’s disease.

Being a G-protein-coupled receptor, HCA_1_ is presumably a good target for drugs; more than 30% of all drugs approved by the FDA today mediate their action through G-protein-coupled receptors [36]. HCA_1_ agonists, including L-lactate, therefore, represent a promising new therapy in stroke and can be given in a time window where no current treatments are available. Although L-lactate in this study was distributed by i.p. injection, L-lactate is an endogenous substance; hence, it is relatively well-tolerated. Some studies show an anxiety-inducing effect in vulnerable individuals [37]. The anxiety-inducing effect appears to be dependent on a rapid infusion of large doses of L-lactate. It is suggested to be caused by weak calcium chelation and subsequent depolarization of neurons. Nevertheless, infusion of L-lactate, in the form of ringer-lactate, is used to replace electrolytes and fluid in patients with low blood volume, hypotension or metabolic acidosis. Lactate, hence, represents a cheap and relatively safe treatment, and the translation of our results into human studies of L-lactate is intriguing. 

## 4. Materials and Methods

### 4.1. Animals

The HCA_1_ knockout (KO) line was generated as described [38]: by homologous recombination in embryonic stem cells, the exon encoding murine HCA_1_ was replaced by the genes for β-galactosidase (LacZ) and neomycin resistance. The mice were a gift from Professor Stefan Offermanns, Max Planck Institute for Heart and Lung Research, Bad Nauheim, Germany, and were backcrossed into a C57Bl/6N background nine times after arriving in our lab. The mice were stalled at the Section of Comparative Medicine at the Institute of Basic Medical Sciences, Faculty of Medicine, University of Oslo with access to food and water ad libitum, and were stalled in a 12:12 h light:dark cycle. The cages had environment enrichments (a plastic house or toilet roll core along with paper for nest building, but no running wheel). The in vivo experiments described in this study were approved by the Norwegian Animal Use and Care Committee (FOTS #14204, approval date 19 April 2018; FOTS #12521, approval date 16 June 2017) and reported in compliance with the Animal Research: Reporting in Vivo Experiments (ARRIVE) guidelines, version 2.0 [39]. All experiments involving live animals were conducted in strict accordance with the national and regional ethical guidelines, and animals were treated by Federation of Laboratory Animal Science Association (FELASA) certified personnel. 

### 4.2. Permanent Occlusion of the Distal Medial Cerebral Artery

At three months of age, stroke was induced in the mice by permanent coagulation of the distal middle cerebral artery (dMCA), as described [27]. The mice were deeply anaesthetized (4% isoflurane, ~70% N_2_O, 30% O_2_) and received buprenorphine 0.3 mg/kg, i.p. (Temgesic, Indivior, Richmond, VA, USA) for analgesia. During the surgery, the body temperature of the mice was maintained at 37 °C and the mice were kept in deep anesthesia (1.5% isoflurane). A small incision of the skin was made, and the temporal muscle was partly detached to allow visual identification of the dMCA through the transparent skull. The dMCA was accessed via careful craniectomy and coagulated (VIO 50C, Erbe, Tübingen, Germany) both proximally and distally to the first bifurcation downstream of the lenticulostriate arteries (M1 to M2). After 30 s, the artery was gently touched with blunted forceps to check for reperfusion. At any sign of reperfusion, the electrocoagulation was repeated. The temporal muscle was placed back to its position and the incision wound was sutured. The mice were placed in a nurturing box at 35 °C to recover from the anesthesia before they were moved back to their home cage. Buprenorphine 0.1 mg/kg i.p. was administered 24 h after the operation and daily thereafter for four days to provide postoperative analgesia. Sham operations were performed on three mice as a procedure control [27]. Sham operations included the entire procedure described above, including craniectomy, but excluding coagulation of the artery. 

### 4.3. L-Lactate and Saline Injections

HCA_1_ KO or WT mice (both sexes; about equal male/female distribution) were randomized into two treatments: sodium L-lactate injections or saline injections (control); sacrificed at one week or three weeks after stroke. The animals treated with L-lactate received two i.p. injections of sodium L-lactate (2 g/kg body weight; 200 mg/mL dissolved in 0.9% saline; pH adjusted to 7.4; i.e., 18 mmol/kg), at 24 h and 48 h after stroke induction. The saline-treated control mice received the same volume (per kg body weight) of 0.9% saline. The mice were sacrificed at one week (WT saline, n = 6; WT L-lactate, n = 9; KO saline, n = 9; KO L-lactate, n = 11) or three weeks (WT saline, n = 10; WT L-lactate, n = 10; KO saline, n = 8; KO L-lactate, n = 10) after the dMCAO.

### 4.4. Exclusion Criteria

Exclusion criteria were set a priori and described in the animal welfare protocol (FOTS 14204). These were as follows: at any sign of distress, e.g., weight loss of more than 10% in one week or more than 15% during the three weeks after stroke, stereotypical behavior, lack of grooming, etc., the animal was withdrawn from the study. During surgery, animals who experienced arterial bleeding or spontaneous recanalization more than once were excluded from the study. Following the dMCAO surgeries, six male WT, five female WT, one male KO, and one female KO mouse died prior to day seven, most of them during the first day. These were not included in this study. The female WT mice who died were all housed in the same cage; therefore, their premature death may have resulted from a malfunctioning cage or other causes unrelated to the treatment. At the time of analysis, mice who showed stroke injuries > 20 mm^3^, that were not restricted to cortical regions—for instance, including part of the underlying striatum—were excluded from the analyses. The same was true for mice where the total stroke volume was <3 mm^3^. These were as follows: after one week: WT saline, n = 1; WT lactate, n = 1; KO saline, n = 3; KO lactate, n = 4. After three weeks: WT saline, n = 1; WT lactate, n = 2; KO lactate, n = 1. In the cases where the lesion volume was >20 mm^3^, there were often signs of a secondary cerebral hemorrhage and the lesion extended across the corpus callosum and into the ipsilateral striatum (one week: WT lactate, n = 1; KO lactate, n = 1). 

### 4.5. Tissue Preparation and Analysis of Stroke Volume

At one or three weeks after dMCAO induction, the mice were anesthetized by i.p. injection of zolazepam 3.3 mg/mL, tiletamine 3.3 mg/mL, xylazine 0.5 mg/mL, and fentanyl 2.6 μg/mL; 0.1 mL/g bodyweight. The deeply anesthetized mice were transcardially perfused with 4% formaldehyde in 0.1 M sodium phosphate buffer; pH 7.4. After perfusion, the brain was gently removed from the skull, post-fixated in 4% PFA at 4 °C overnight, then transferred to a 0.4% PFA solution and kept at 4 °C. The brains were then allowed to sink in 30% sucrose overnight for cryoprotection before serial cryosections of 20 µm thickness from the entire brains were produced at −20 °C (Thermo Scientific™ HM 450 Sliding Microtome, Waltham, MA, USA). The sections were stored in chronological order in separate wells filled with 5 mL 0.1 M NaPi buffer with 0.05% sodium azide, allowing full control of the sequence of the serial sections. 

### 4.6. Staining with Cresyl Violet

Every 8th section was mounted on 25 × 75 × 1.0 mm glass slides (Superfrost Plus™ from Thermo Scientific™, Waltham, MA, USA) and stained with CV as follows: EtOH 95% (15 min); 70% (1 min); 50% (1 min) in phosphate-buffered saline (PBS), PBS (2 min + 1 min), incubation with filtered CV (1 g/L) at 60 °C; 8 min, PBS (2 × 2 min), 95% EtOH (1 min), 1% glacial acetic acid in 95% EtOH (3 s), 95% EtOH (5 s) Neo-Clear (Merck, Darmstadt, Germany, 1 min), mounted with Neo-Mount (Merck, Darmstadt, Germany) and coverslipped.

### 4.7. Imaging and Lesion Volume Measurements

Surface images of the CV-stained coronal sections were obtained at 20× magnification (Axio Scan Z1, Carl Zeiss Microscopy, Oberkochen, Germany; SteREO Lumar.V12, Carl Zeiss Microscopy, Oberkochen, Germany). Images were analyzed using FIJI (Image J, version: 2.0.0-rc-69/1.52i). The analyses were performed by observers who were blinded to the treatments and genotypes. Since the lesion produced by dMCAO is restricted to the cortical areas, and atrophy is pronounced at 3 weeks after exercise, we could not measure the lesion area directly at this time point. Instead, the lesion area was measured as follows: the cortex of the ipsilateral (to the stroke) and contralateral hemisphere was outlined according to The Allen Brain Atlas (https://mouse.brain-map.org/experiment/thumbnails/100048576?image_type=atlas (accessed on 31 May 2019)). The lesion area in each section was calculated by subtracting the cortical area of the ipsilateral hemisphere (excluding any visibly damaged and/or scarred tissue) from the cortical area of the contralateral hemisphere in each section (Swanson Method [40]). This was conducted for every section at 160 µm intervals between 1.5 mm rostral and 2.5 mm dorsal of bregma forming the base to calculate total lesion volume. In order to keep compatibility between the datasets, we analyzed the brain sections from the mice obtained at one week after stroke in the same manner (but the actual lesion area is outlined in Figure 2 for visualization). 

### 4.8. Immunohistochemistry

From each animal, one 20 µm coronal brain section (bregma −0.245) was mounted on microscope slides (Superfrost Plus, Thermo Scientific, Waltham, MA, USA) and left to dry. Antigen retrieval: either the sections were rinsed in PBS and exposed to freshly made pepsin solution (10 mg/mL, diluted in 0.2 M HCl) at 37 °C for 20 min to increase the antibody penetration of the basal laminae to allow proper collagen IV staining (used for capillary density measurements) or the sections were incubated in citrate buffer (0.1 M, pH 8.6) at 80 °C for 30 min (Ki-67 and nestin, neurogenesis markers). The sections were then rinsed 3 × 10 min in PBS (collagen IV) or 2 × 10 min (Ki-67 and nestin) and then exposed to a blocking solution (10% fetal calf serum and 0.5% triton x-100) for two hours before being exposed to the primary antibodies (rabbit anti-collagen IV; Abcam ab6586 diluted 1:500 or mouse anti-nestin, Abcam, ab6142, Cambridge, UK; diluted 1:500, and rabbit anti-Ki-67, Abcam, ab15580, Cambridge, UK; diluted 1:500) in blocking solution overnight. The following day, the sections were rinsed in PBS for 6 × 10 min before they were incubated for two hours with anti-rabbit Alexa Fluor 488 (IgG, catalogue #A21206, diluted 1:500 in blocking solution) or anti-mouse Alexa Fluor 488 (A21202, Invitrogen, Carlsbad, CA, USA; diluted 1:1000 in blocking solution) and anti-rabbit Alexa 647 (A27040, Invitrogen, Carlsbad, CA, USA; diluted 1:1000 in blocking solution). The sections were then rinsed in PBS 3 × 10 min, before incubation with DAPI (4′,6-diamidino-2-phenylindole, D9542, Sigma-Aldrich, St. Louis, MO, USA; stock solution 1 mg/mL diluted 1:5000 in PBS) at room temperature for 15 min. Finally, the sections were rinsed in PBS 3 × 10 min and coverslipped with ProLong Gold (Thermo Fisher Scientific, Waltham, MA, USA).

### 4.9. Image Acquisition and Quantification of Capillaries

Z-stack images of the whole coronal brain sections (20 µm optical thickness) were obtained at 20× magnification using an automated slide scanner system (Axio Scan Z1, Carl Zeiss Microscopy, Oberkochen, Germany). Images were analyzed using Fiji (version 2.0.0-rc-69/1.52p; Java 1.8.0_172). Capillaries were outlined using a semi-automated method, adapted with the trainable Weka segmentation (TWS) plug-in and the capillary density was given as the area covered by collagen IV-stained capillaries as percentage of a defined ROI. On the ipsilateral side, the ROI was defined as the cortical area surrounding the infarct core by 500 μm in case there was an obvious lesion visible, otherwise the cortical area in the assumed lesion center was measured, as outlined in Figure 3. Similarly, the contralesional ROI was defined as the symmetrical position to the stroke lesion, constituting a surrounding area of 500 μm. Vessels above 10 µm in diameter were excluded from the ROI. All measurements were limited to only include the cortex and were performed by an observer who was blinded to the genotypes and treatments.

### 4.10. Image Acquisition and Quantification of the Neurogenesis Markers Ki-67 and Nestin

Z-stack images of 0.5 µm optical thickness for each section of the stroke area on the coronal brain sections from animals sacrificed one week after stroke were obtained at 20× magnification (Andor Dragonfly spinning disk confocal microscope, Oxford Instruments, Abingdon, UK). Images were analyzed using Fiji (version 2.1.0/1.53c; Java 1.8.0_172). The lesion site was used as the basis to determine the dorsal and ventral border of the ROI (Figure S3). The corpus callosum border towards the striatum was used as the medial border of the ROI and the lateral border was set 200 μm from the medial border. For the animals sacrificed at three weeks post-stroke, a larger ROI was defined, constituting a medial border (1700 µm along the corpus callosum border) and a lateral border from the medial border to the cortical lining. Ki-67-positive cells were quantified as the number of DAPI-positive nuclei that co-expressed Ki-67. The high density of the nestin-positive cells made it unreliable to count the nestin-positive cells manually; therefore, nestin-positive cells were quantified by thresholding: nestin expression was determined as the area of nestin labeling above background levels of the total ROI. All measurements were performed by an observer who was blinded to the genotypes and treatments.

### 4.11. Statistics

The test of homogeneity of variances (IBM SPSS vs26) resulted in a *p*-value > 0.05 for all analyses except for the neurogenesis markers (nestin and Ki-67). The groups were compared using one-way ANOVA with Tukey post hoc or non-parametric Kruskal–Wallis test as appropriate (IBM SPSS vs26). A *p*-value below 0.05 was defined as statistically significant for all tests. 

## Data Availability

The datasets generated and/or analyzed during the current study are available from the corresponding authors upon reasonable request.

## Supplementary Materials

The supporting information can be downloaded at: https://www.mdpi.com/article/10.3390/ijms25021232/s1.
