# Supplementary material for: L-Lactate Treatment at 24 h and 48 h after Acute Experimental Stroke Is Neuroprotective via Activation of the L-Lactate Receptor HCA1
Source: Int J Mol Sci. 2024 Jan 19;25(2):1232. doi: 10.3390/ijms25021232 (PMC10816130; doi:10.3390/ijms25021232)
Supplement: Supplementary file 1 [file ijms-25-01232-s001.zip › ijms-2812700-supplementary.pdf]

SUPPLEMENTARY FIGURES

Figure S1

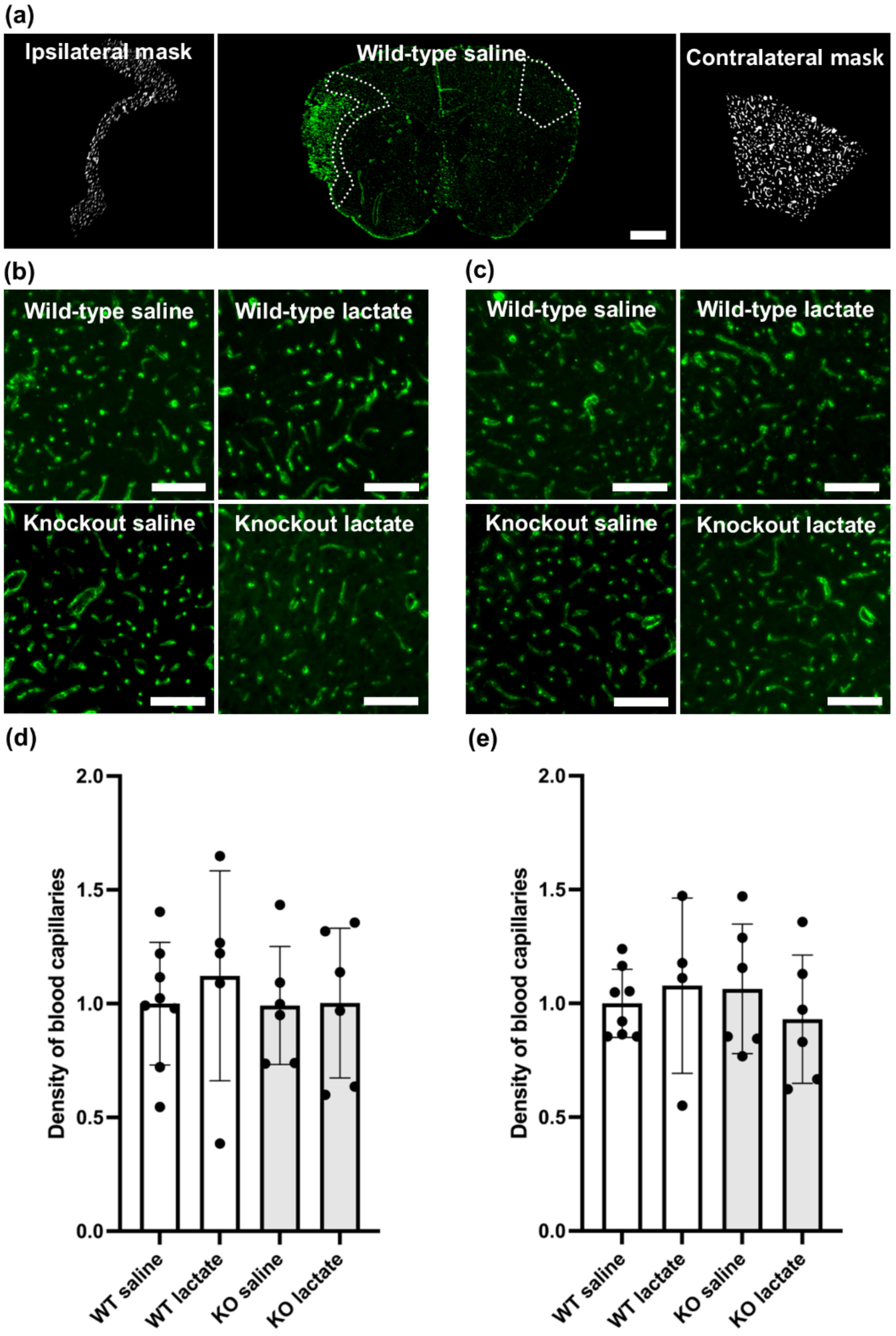

Figure S1. HCA_1-dependent angiogenesis 1 week after stroke**.** (**a**) Coronal section immunolabeled for the basal lamina marker collagen IV (green) in the middle; the selections used to quantify capillary densities is shown by the dotted lines, and the resulting masks are shown on each side. (**b**) Representative confocal images of capillaries in the ipsilateral cortex of all treatment groups. (**c**) Representative confocal images of capillaries in the ipsilateral cortex of all treatment groups. (**d**) Quantitative assessment of the capillary densities of the ipsilateral cortex of WT mice (white bars) and HCA_1 KO mice (grey bars) after treatment with saline or lactate. Numbers represent the area covered by capillaries divided by the area of the region of interest (ROI; area covered by larger vessel subtracted) and are normalized to the average capillary density of the WT control (mean±SD). (**e**) Quantitative assessment of the capillary densities of the contralesional cortex of WT mice (white bars) and HCA_1 KO mice (grey bars) after treatment with saline or lactate. Numbers represent the area covered by capillaries divided by the area of the region of interest (area covered by larger vessel subtracted) and are normalized to the average

capillary density of the WT control (mean±SD). The black dots represent individual mice. scale bars = 1 mm (**a**) and 125 μm (**b**-**c**). and contralateral.

Figure S2

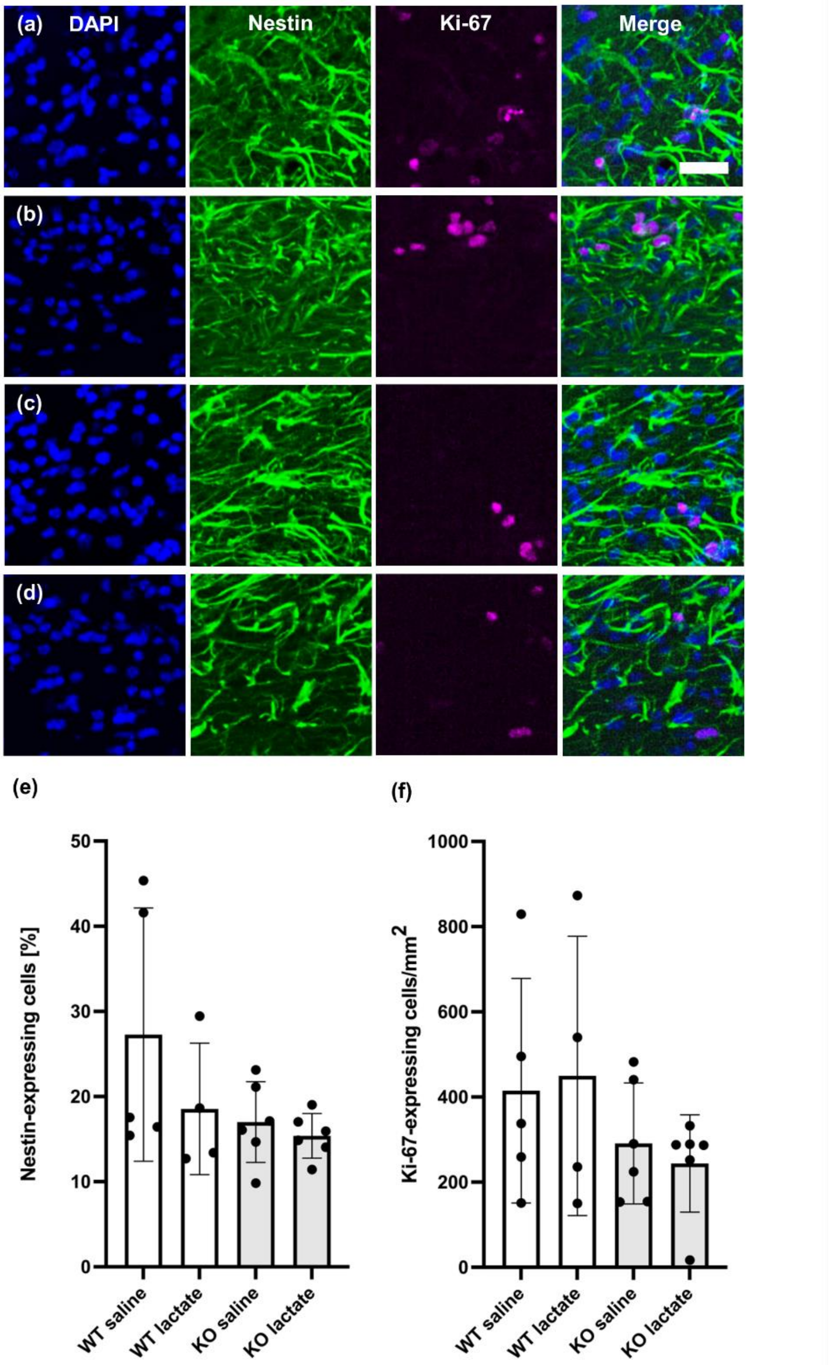

Figure S2: Neurogenesis or neural migration observed at 1 weeks after stroke was not affected by lactate treatment. Coronal section immunolabeled with markers for neuroprogenitor cells (nestin; green), proliferating neuroblasts (Ki-67; magenta) and nuclei (DAPI; blue), of WT mice treated with (**a**) or saline or (**b**) L-lactate, and HCA_1 KO mice treated with (**c**) saline or (**d**) L-lactate. Enhanced neurogenesis or neural migration to the lesional/perilesional site did not differ between the groups at this time point. **(e-f):** Quantitative assessment of the neuroprogenitor cells (**e**) and proliferating neuroblasts (**f**) of WT mice (white bars) and HCA_1 KO mice (grey bars). The black dots represent individual mice. Scale bar = 33 μm.

Figure S3

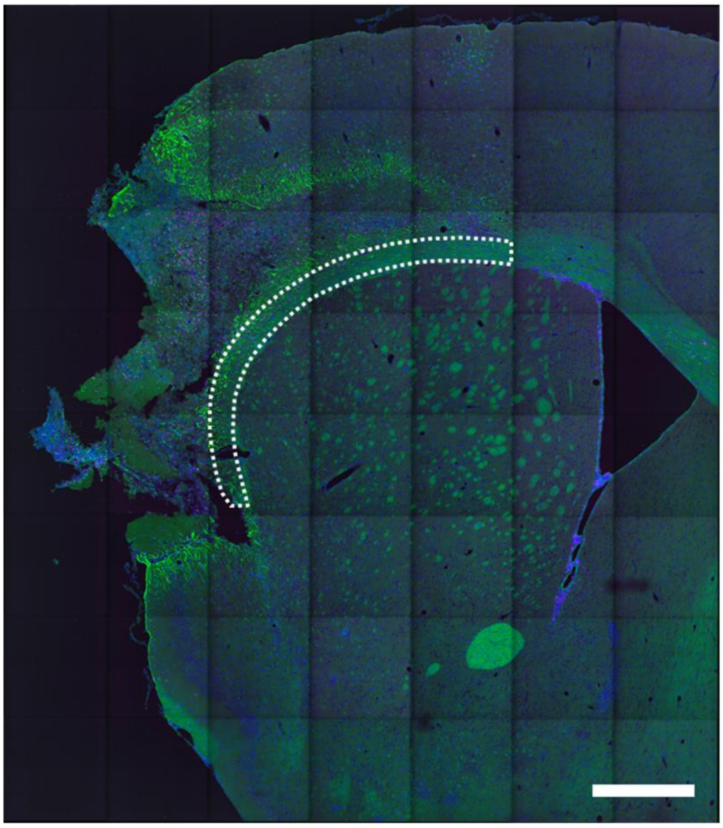

Figure S3: Example of ROI for quantitative assessment of neuroprogenitor cells. Coronal section from a one-week post-stroke control mice, immunolabeled with DAPI (blue), nestin (green) and Ki-67 (magenta), showing the ROI (stitched line) used to quantify neuroprogenitor cells. Scale bar = 500 µm.
